# Hsp70 regulates CD24 expression and promotes metastasis and invasion of lung cancer via the MAPK/ERK signaling pathway

**DOI:** 10.3389/fonc.2025.1665342

**Published:** 2025-10-21

**Authors:** Xin Liu, Xinjing Yu, Qun Hu, Ning Zhang, Shuyu Zhang, Yang Liu, Jiaqi Jia, Xiaojuan Qiao

**Affiliations:** ^1^ The Affiliated Hospital of Inner Mongolia Medical University, Inner Mongolia Medical University, Hohhot, China; ^2^ Department of Medical Oncology, The Affiliated Hospital of Inner Mongolia Medical University, Inner Mongolia Medical University, Hohhot, China; ^3^ Central Laboratory, Bayannur Hospital, Bayannur, Inner Mongolia, China; ^4^ School of Arts and Sciences, University of Rochester, Rochester, NY, United States; ^5^ Graduate School of Youjiang Medical University for Nationalities, Youjiang, Baise, China

**Keywords:** Hsp70, CD24, lung cancer, MAPK/ERK pathway, mechanism & characterization

## Abstract

**Purpose:**

To investigate the effects and mechanisms of Hsp70-mediated regulation of CD24 expression on the invasion and metastasis of lung cancer.

**Methods:**

Protein-protein interactions between Hsp70 and CD24 were analyzed by co-immunoprecipitation and immunofluorescence. Lentiviral-mediated Hsp70 knockdown and CD24 overexpression were validated using RT-qPCR and Western blotting. Functional consequences were assessed through CCK-8 proliferation assays, colony formation, wound healing, Transwell migration/invasion, and angiogenesis assays. *In vivo* metastatic potential was evaluated using a tail vein injection model in nude mice.

**Results:**

Hsp70 and CD24 demonstrated interaction through co-precipitation. Knockdown of Hsp70 significantly attenuated CD24 expression (p<0.01), whereas CD24 overexpression did not alter Hsp70 levels. Phenotypically, Hsp70 suppression impaired cellular proliferation, migration, invasion and angiogenic capacity, while CD24 overexpression potentiated these oncogenic properties. Both manipulations modulated ERK1/2, MEK, Raf and Ras phosphorylation status.

**Conclusion:**

Hsp70 upregulates CD24 expression and activates downstream MAPK/ERK signaling, thereby enhancing the metastatic and invasive capacity of lung cancer.

## 1 Introduction

Lung cancer remains one of the most prevalent malignancies worldwide, representing a significant global health burden. Epidemiological data reveal it as the predominant cause of cancer-related mortality in males and the second leading cause in females, surpassed only by breast carcinoma (1). Although diagnostic and therapeutic modalities have advanced considerably in recent years, the prognosis for patients with advanced or metastatic disease remains dismal, demonstrating a 5-year survival rate of merely 15-30% (2).

The current situation regarding the diagnosis and treatment of lung cancer remains severe. We found that heat shock protein 70 (Hsp70) and cluster of differentiation 24 (CD24) are highly expressed in lung cancer tissues and cells. These two interacting proteins exhibit a direct or indirect interaction and are associated with poor prognosis in lung cancer (3). They can serve as biomarkers for assessing lung cancer prognosis and hold potential as therapeutic targets.

Hsp70 is a highly conserved protein produced under stress conditions and serves as a crucial molecular chaperone. It plays diverse roles throughout cellular processes, functioning in nearly all stages of the lifecycle of proteins from synthesis to degradation. These roles include folding newly synthesized proteins, facilitating the translocation of peptides to mitochondria and endoplasmic reticulum, regulating protein activity, and disassembling protein complexes (4, 5). Under normal conditions, Hsp70 prevents the aggregation of misfolded proteins and promotes their refolding and the dissolving of aggregated proteins, while it coordinates with cellular degradation mechanisms to clear abnormal proteins and protein aggregates (6–8).

Multiple studies have indicated that there are elevated levels of Hsp70 expression in many types of cancer and that these elevated levels correlate with poor prognosis, recurrence, and drug resistance (9–11). In certain rodent models, overexpression of Hsp70 has been associated with increased tumor growth and metastatic potential, while Hsp70 knockdown has resulted in tumor shrinkage, even leading to complete tumor regression (12). The pro-oncogenic functions of Hsp70 operate through both intracellular and extracellular mechanisms. Within cells, Hsp70 enhances tumor cell survival, treatment resistance, and malignant progression by suppressing multiple apoptotic pathways—such as inhibiting Bax activation and the formation of the Apaf-1/caspase-9 apoptosome—stabilizing lysosomal membranes, promoting autophagy, and modulating key oncogenic signaling pathways including PI3K/Akt, NF-κB, and MAPK/ERK. Extracellularly, Hsp70 can be actively secreted or passively released from necrotic cells (13, 14). As a damage-associated molecular pattern (DAMP), extracellular Hsp70 acts as an endogenous “danger signal” capable of activating immune cells via receptors such as Toll-like receptors (TLR2/4). However, in the context of cancer, it is more frequently reported to contribute to an immunosuppressive tumor microenvironment by mediating immune evasion and facilitating tumor progression and metastasis (15, 16). Based on existing research reports and corroborating preliminary studies from our research group (3, 17), CD24 can directly or indirectly interact with Hsp70 and is associated with patient prognosis, potentially serving as one of the client proteins that binds to Hsp70.

CD24, also known as heat-stable antigen (HSA), is a highly variable glycosylphosphatidylinositol-anchored membrane protein expressed at high levels in various tumors and is correlated with patient prognosis. Because it lacks intracellular structure, CD24 cannot directly transmit transmembrane signals, but it can utilize lipid rafts in the cell membrane as platforms for transmembrane signal transduction (18). In recent years, increasing evidence has supported the cancer stem cell theory, which suggests that tumor recurrence and metastasis originate from the activation of cancer stem cells. CD24 is also considered a marker of cancer stem cells, with its expression level being closely associated with tumor invasion, metastasis, and prognosis (19–21). Our research group has confirmed that CD24 and Hsp70 jointly affect the prognosis of lung cancer patients. However, their impact on lung cancer invasion and metastasis, as well as the underlying mechanisms, remains unclear. To address this issue, our research team has carried out the following research.

## 2 Materials and methods

### 2.1 Cell lines and cell culture

The human lung cancer cell lines A549, H1975, and H1650 used in this experiment were all obtained from Shanghai Yaji Biotechnology Co., Ltd. Cells were cultured in DMEM medium(Gibco, USA) supplemented with 10% fetal bovine serum(Gibco, USA) and 1% penicillin/streptomycin(Gibco, USA) at 37°C in a humidified atmosphere containing 5% CO_2_.

### 2.2 Lentiviral transduction

Lentiviral transduction of Hsp70 knockdown-cDNA was performed in H1975 cells, while lentiviral transduction of CD24 overexpression-cDNA was carried out in A549 cells. The plasmids(Shanghai Genechem Co.,Ltd.) used in the experiment include: pLVX-shRNA2, a short hairpin RNA vector targeting the human HSP70 gene; and pLVX-IRES-ZsGreen1, an overexpression vector carrying the complete coding sequence (CDS) of the human CD24 gene. Seed HEK293T cells in logarithmic growth phase into a 10 cm culture dish. At 80% confluence, transfect using Lipofectamine™ 3000 according to the manufacturer’s instructions. Replace with fresh medium 6–8 hours post-transfection and continue culture. Viral supernatants were collected at 48 h and 72 h post-transfection. After filtration through 0.45 μm PVDF membranes to remove cellular debris, the supernatants were concentrated by ultracentrifugation. The resulting viral pellets were resuspended in pre-chilled PBS, aliquoted, and stored long-term at −80 °C.

### 2.3 Quantitative polymerase chain reaction

Total RNA was extracted from H1975 and A549 cells using the RNA Extraction Kit(Vazyme, China). The extracted total RNA was reverse transcribed using the Reverse Transcription Kit(Vazyme, China). The resulting cDNA was mixed with PCR reagents(Vazyme, China) and subjected to quantitative PCR using the Thermo 7500 Fast Real-Time PCR System. The program settings were as follows: an initial denaturation stage at 95°C for 30 seconds, followed by 40 cycles of denaturation at 95°C for 10 seconds and annealing/extension at 60°C for 30 seconds. The melting curve stage was set to default. GAPDH was used as the reference gene for normalization, and each experiment was repeated three times. The PCR primers are shown in **Supplementary Table S1**.

### 2.4 Protein extraction and western blot

Total cellular proteins were extracted using the Beyotime Protein Extraction Kit. Cells were harvested at 80–90% confluence, centrifuged, and then mixed with RIPA lysis buffer (P0013B, Beyotime, China) on ice for 30 minutes, with mixing every 10 minutes. After centrifugation at 12,000g for 15 minutes, the protein samples were mixed with SDS-PAGE loading buffer (P0015, Beyotime, China) and heated to 95°C for 10 minutes. The protein samples were loaded onto polyacrylamide gels for electrophoresis and then transferred onto PVDF membranes. After blocking with milk at room temperature for 2 hours, the membranes were incubated overnight at 4°C with primary antibodies against HSP70, CD24, and GAPDH, respectively. The membranes were then washed three times with TBST and incubated with corresponding secondary antibodies at room temperature for 1 hour. After washing with TBST again, and protein bands were visualized. Images were captured, and protein expression levels were analyzed. Each experiment was repeated three times. All antibodies used in this study are provided in **Supplementary Table S2**.

### 2.5 Cell counting kit-8 cell proliferation assay

Cells were seeded into 96-well plates at a density of 5×10^3^ cells per well. Equal volumes of CCK-8 reagent were added at 24, 48, and 72 hours, and the plates were then incubated at 37°C for 2 hours. The absorbance of cells at a wavelength of 450nm was measured using a microplate reader, and the data were recorded and analyzed. Each experiment was repeated three times.

### 2.6 Cell migration and invasion assay

Cells from each group were mixed with 200μL serum-free culture medium and seeded into chambers with or without matrix gel at a density of 5 × 10^4^ cells per chamber. Chambers with matrix gel were used for invasion assays, while those without matrix gel were used for migration assays. The chambers were placed in a 24-well plate with 600μL of medium containing 20% FBS in the lower chamber. After 24 hours, the upper surface of the chambers was gently wiped with a cotton swab, fixed with methanol for 20 minutes, and then stained with 0.1% crystal violet solution for another 20 minutes. After washing, images were captured, and cells were counted for analysis. Each experiment was repeated three times.

### 2.7 Clonogenic assay

Cells from each group were seeded into 6-well plates at a density of 1×10^3^ cells per well. After incubation for 14 days, the cells were fixed with methanol for 20 minutes and then stained with 0.1% crystal violet solution for another 20 minutes. Photographs were taken, and clonogenic cells were counted for analysis. Each experiment was repeated three times.

### 2.8 Wound-Healing assay

Seed 3×10^5^ cells per well in a 6-well plate. On the second day, when cells reach 90% confluence, a UV-treated ruler was placed perpendicular to the plate, and a scratch was made along the ruler using a 100μL pipette tip. Consistent force was applied during the scratching process. After scratching, the medium was removed, and the cells were washed with PBS to remove cell debris. Fresh medium was added, and photographs were taken. The cells were then incubated for another 24 hours before taking photographs again and performing cell counting analysis. Each experiment was repeated three times.

### 2.9 Angiogenesis assay

Supernatants from each group of cells were collected and filtered through a 0.22μm filter to maintain sterility and stored at 4°C. 9-day-old fertilized chicken embryos were windowed at the air sac end (blunt end) under sterile conditions, removing the air sac membrane to expose the chick embryo chorioallantoic membrane chick embryo chorioallantoic membrane(CAM). Sterile gelatin sponges sized 2×2×2mm^3^ were soaked in the respective cell supernatants and placed at sparse vascular regions of the CAM. The window was sealed with sterile transparent tape, and the embryos were further incubated in a humidified chamber at 38.5°C to 39°C and 65% to 70% relative humidity. After 4 days, the transparent tape was carefully removed to expose the CAM, and photographs were taken to observe neovascularization at the gelatin sponge sites. Each experiment was repeated three times.

### 2.10 Immunofluorescence

Cells were seeded into pre-treated coverslips in a 24-well plate and cultured until reaching near confluency. The coverslips were then removed, and cells were washed three times with PBS. They were fixed with methanol for 20 minutes and then incubated with 0.5% Triton for 20 minutes. After washing with PBS, they were incubated with BSA at room temperature for 30 minutes. Subsequently, they were air-dried, and primary antibodies were added and kept at 4°C overnight. The following day, fluorescent secondary antibodies were added and incubated in the dark for 1 hour. Observation and photographing were done under a fluorescent microscope, and analysis was conducted. Each experiment was repeated three times.

### 2.11 Immunoprecipitation

Firstly, total cellular proteins were extracted, and a small amount of lysis buffer was used as a positive control. The remaining lysate was then incubated with 1μg of the target protein overnight at 4°C. Subsequently, 10 μl of protein A/G magnetic beads (#P2179S, Beyotime, China) were washed three times with an appropriate buffer and centrifuged at 1,000×g for 3 minutes. After centrifugation, the magnetic beads were slowly agitated at 4°C for 2–4 hours. The protein A/G magnetic beads were collected in a magnetic rack and washed 3–4 times with lysis buffer. After washing, 15μl of SDS loading buffer (2×) was added, and the mixture was boiled for 5 minutes before performing SDS-PAGE and protein blotting experiments. Each experiment was repeated three times.

### 2.12 Tail vein lung metastasis assay

Twenty-seven 5-week-old male SPF BALB/c nude mice weighing 17 ± 2 g were housed in the SPF animal facility at Inner Mongolia Medical University. The feed, water, and bedding were sterilized strictly. Throughout the experiment, humanitarian care was provided according to the principles of the 3Rs of animal experimentation. After one week of acclimatization, the experiment was conducted. The lung cancer cells from the knockout and overexpression groups were prepared into cell suspension with a density of 1x10^6^ cells/ml (n = 6 per group). Mice were restrained on the operation table, and after disinfection, 200 μL of cell suspension was injected into the tail vein. Control and experimental group mice were dissected at the 5th, 6th, and 7th weeks post-injection. Lung lesions, pleural effusion formation, tumor and nodule counts, and observations of metastasis in other organs were recorded and analyzed.

### 2.13 Flow cytometry

Flow cytometry was employed to detect the expression levels of lentiviral fluorescent transfections in cells. Test cells were trypsinized to prepare a single-cell suspension, washed with pre-chilled PBS, and resuspended. Prior to analysis, filter the cell suspension through a 70 μm nylon mesh to remove aggregates. Use untransfected homologous cells as a negative control to establish detection voltage and positive/negative thresholds on the flow cytometer (Beckman Coulter). Ultimately, acquire at least 10,000 cell events to determine the percentage of fluorescent-positive cells and the fluorescence intensity.

### 2.14 Statistical analysis

One-way analysis of variance (ANOVA) was used for statistical analysis, and T-test was used for comparison between groups. Data are expressed as mean ± standard deviation. Differences were considered statistically significant at P < 0.05. All statistical analyses were performed using GraphPad Prism and ImageJ. More information of the materials and methods is in the **Supplementary Materials**.

## 3 Results

### 3.1 Expression of Hsp70 and CD24 in lung cancer cells and the construction and validation of lentiviral stably transfected cell lines

**Figure 1:**
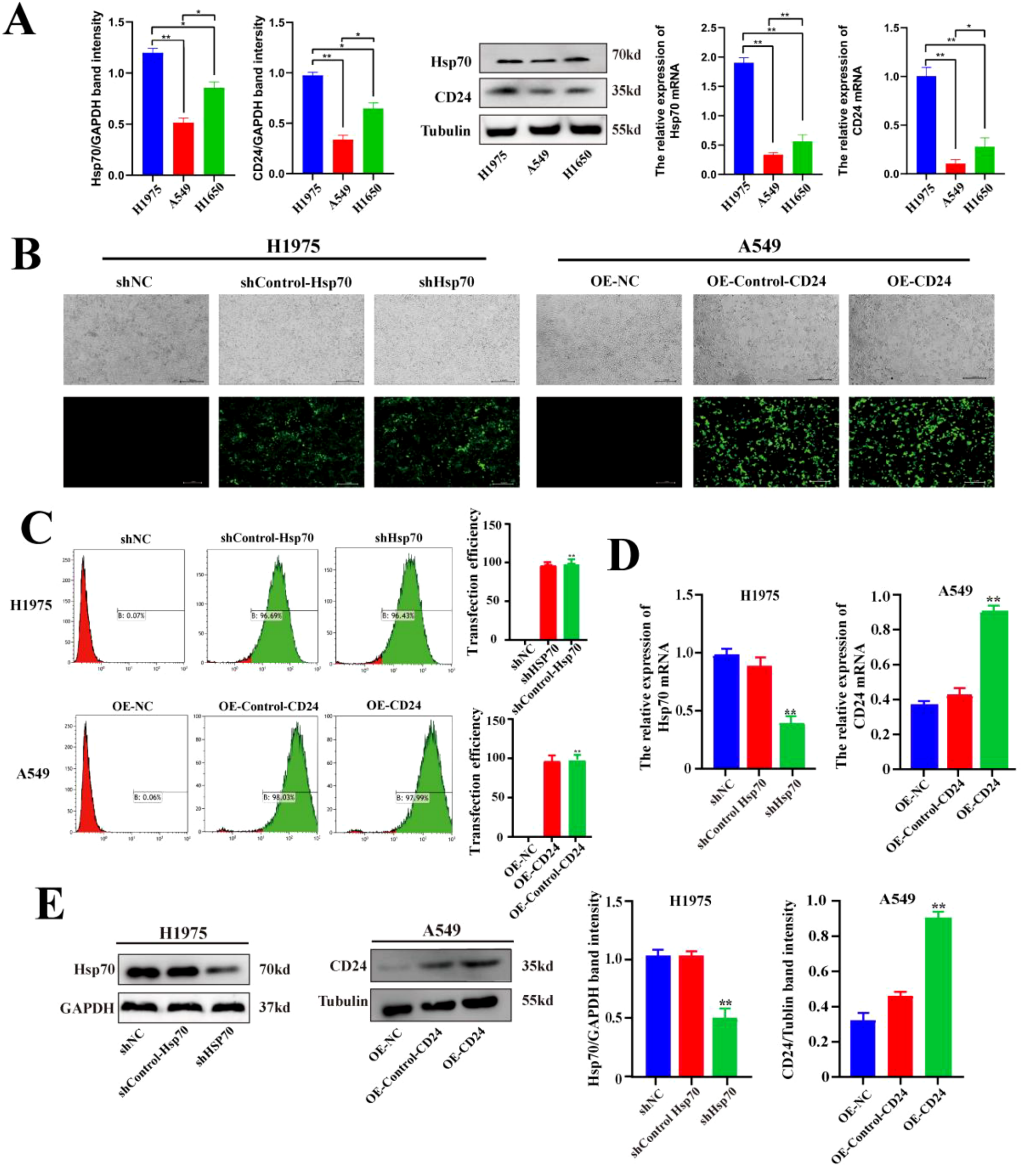
Expression levels of Hsp70 and CD24 in lung cancer cells and the construction and validation of lentiviral stably transfected cell lines. **(A)** Western blot and qPCR assays for the expression levels of Hsp70 and CD24 proteins and their corresponding mRNAs in H1975, A549, and H1650 cells. **(B)** Fluorescence microscopy observation of lentiviral transduction in H1975 and A549 cells. **(C)** Flow cytometry detection of GFP fluorescence signals following lentiviral transfection in H1975 and A549 cells. **(D, E)** qPCR and Western blot experiments verified Hsp70 knockdown in H1975 cells and CD24 overexpression in A549 cells. *P < 0.05, **P < 0.01.

In previous studies conducted by our research group, immunohistochemical analysis was performed on 93 cases of lung cancer and paracancerous tissue (3). The results showed that the expression of Hsp70 and CD24 in lung cancer tissues was significantly higher than that in paracancerous tissue, and high expression of Hsp70 and CD24 suggested a poor prognosis for patients. The WB and qPCR results indicated that the expression of Hsp70 and CD24 are relatively highly expressed in H1975 cells, while there is relatively low expression in A549 cells (p<0.01) (**Figure 1A**). We therefore used lentiviral vectors in H1975 cells with relatively high expression of Hsp70 to construct Hsp70 knockdown stable cell lines. In A549 cells with relatively low expression of CD24, lentivirus was used to construct CD24-overexpressing stable cell lines. Results assessed using fluorescence microscopy (**Figure 1B**) and flow cytometry (**Figure 1C**) both indicated successful transfection. WB and qPCR results (**Figures 1D, E**) showed that compared to the sh-NC group (no treatment) and sh-Control-HSP70 group (carrying empty vector), HSP70 was significantly reduced at both the protein and mRNA levels in the sh-HSP70 group (p<0.01), while CD24 was significantly increased at both the protein and mRNA levels in the OE-CD24 group compared with the OE-NC and OE-Control-CD24 groups (p<0.01). These results confirm successful Hsp70 knockdown in H1975 cells and successful CD24 overexpression in A549 cells.

### 3.2 Knockdown of Hsp70 and overexpression of CD24 altered the malignant biological behavior of lung cancer cells

**Figure 2:**
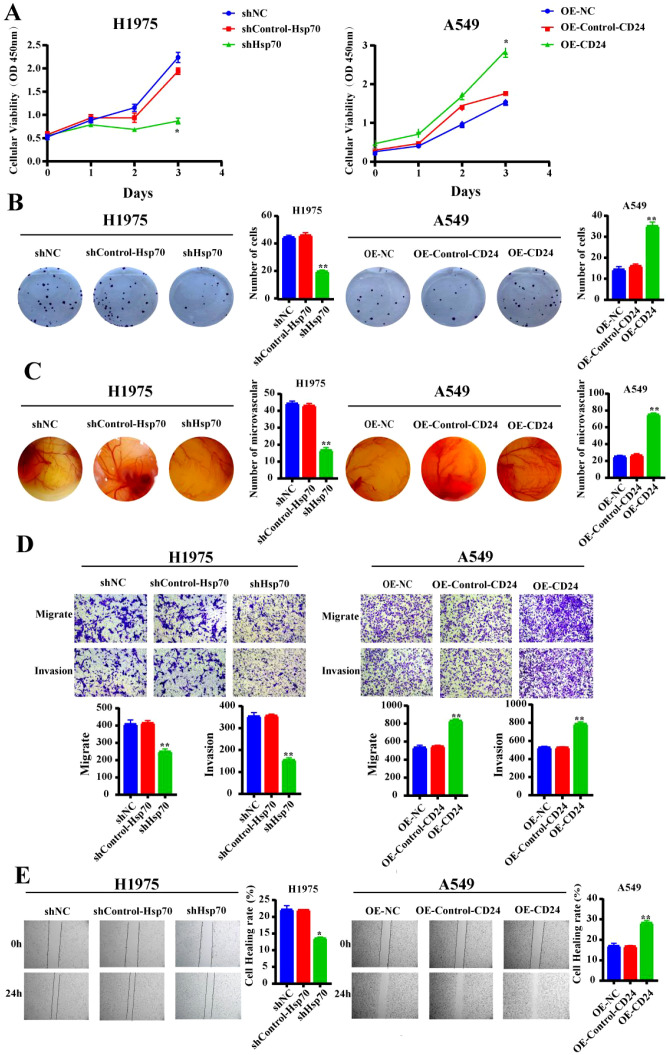
Alteration in malignant biological behavior of lung cancer cells after Hsp70 knockdown and CD24 overexpression. **(A)** The CCK-8 proliferation assay assessed the respective proliferation capabilities of H1975 and A549 cells following Hsp70 knockdown and CD24 overexpression. **(B)** Cloning assay to detect the proliferative capacity of cell lines. **(C)** Use of the chick embryo chorionic allantoic membrane assay to detect effects on blood vessel formation. **(D)** Use the Trans-well assay to show the migratory and invasion capacity *in vitro*. **(E)** Detection of the cell lines migratory capacity using cell scratch assay. *p<0.05; **P<0.01.

To investigate the effects of Hsp70 knockdown and CD24 overexpression on the malignant biological behavior of lung cancer cells, we conducted a series of cellular phenotype experiments. The CCK-8 assay indicated that Hsp70 knockdown significantly reduced the proliferation capacity of H1975 cells compared to the NC and Control groups (p<0.05). All subsequent p-values represent comparisons between the knockdown/overexpression group and the NC and Control groups. Conversely, the proliferation ability of A549 cells was significantly increased after CD24 overexpression (p<0.05) (**Figure 2A**). Colony formation assays demonstrated that Hsp70 knockout resulted in reduced colony number and size in H1975 cells (p<0.01), while CD24 overexpression substantially promoted clonogenic survival in A549 cells (p<0.01) (**Figure 2B**). Angiogenic potential, assessed by CAM assay, was profoundly impaired upon Hsp70 knockdown, as evidenced by decreased vascular density and diameter (p<0.01). Conversely, CD24 overexpression significantly augmented angiogenesis (p<0.01) (**Figure 2C**). The Transwell assay demonstrated that the migratory and invasive abilities of H1975 cells were significantly decreased after Hsp70 knockdown (p<0.01), while overexpression of CD24 increased the migratory and invasive abilities of A549 cells (p<0.01) (**Figure 2D**). Complementary wound healing assays confirmed these findings, with Hsp70 knockdown inhibiting (p<0.01) and CD24 overexpression accelerating (p<0.01) cell migration (**Figure 2E**). Collectively, these findings demonstrate that Hsp70 knockdown suppresses oncogenic phenotypes in lung cancer cells, whereas CD24 overexpression enhances malignant progression.

### 3.3 Overexpression of CD24 partially reverses the malignant biological behavior of lung cancer cells caused by Hsp70 knockdown

**Figure 3:**
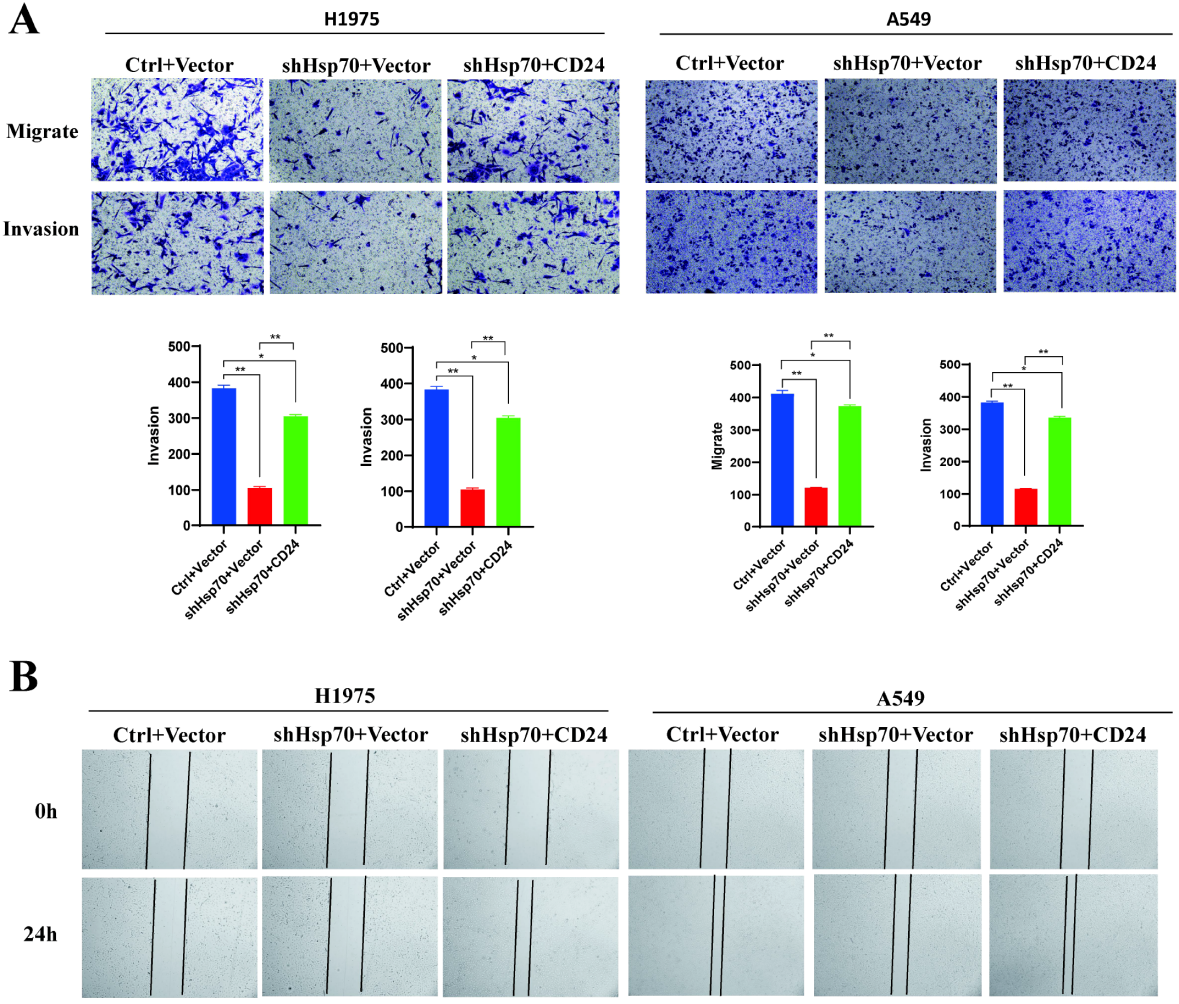
Overexpression of CD24 partially reverses the malignant biological behavior of lung cancer cells caused by Hsp70 knockdown. Ctrl: In the knock-down/knock-out HSP70 experiment, only the empty vector was carried. Vector: In the CD24 expression assay, only the empty vector was carried. shHsp70: Knockdown HSP70. CD24: Overexpression of CD24. **(A)** Trans-well assay. **(B)** Wound-Healing Assay. These experiments demonstrate that knocking down HSP70 alone attenuates cellular proliferation and invasive capacity. However, overexpressing CD24 on top of HSP70 knockdown partially reverses the effects of the former. *P < 0.05, **P < 0.01.

To further investigate the effects of Hsp70 and CD24 on the malignant biological behavior of lung cancer cells, we overexpressed CD24 in H1975 and A549 cells following HSP70 knockdown. Transwell assays and wound-healing assays demonstrated that HSP70 knockout alone impaired cellular proliferation and invasion capacity. However, overexpression of CD24 on the background of HSP70 knockout partially reversed the effects of the former (**Figure 3**).

### 3.4 The interaction between Hsp70 and CD24 in lung cancer

**Figure 4:**
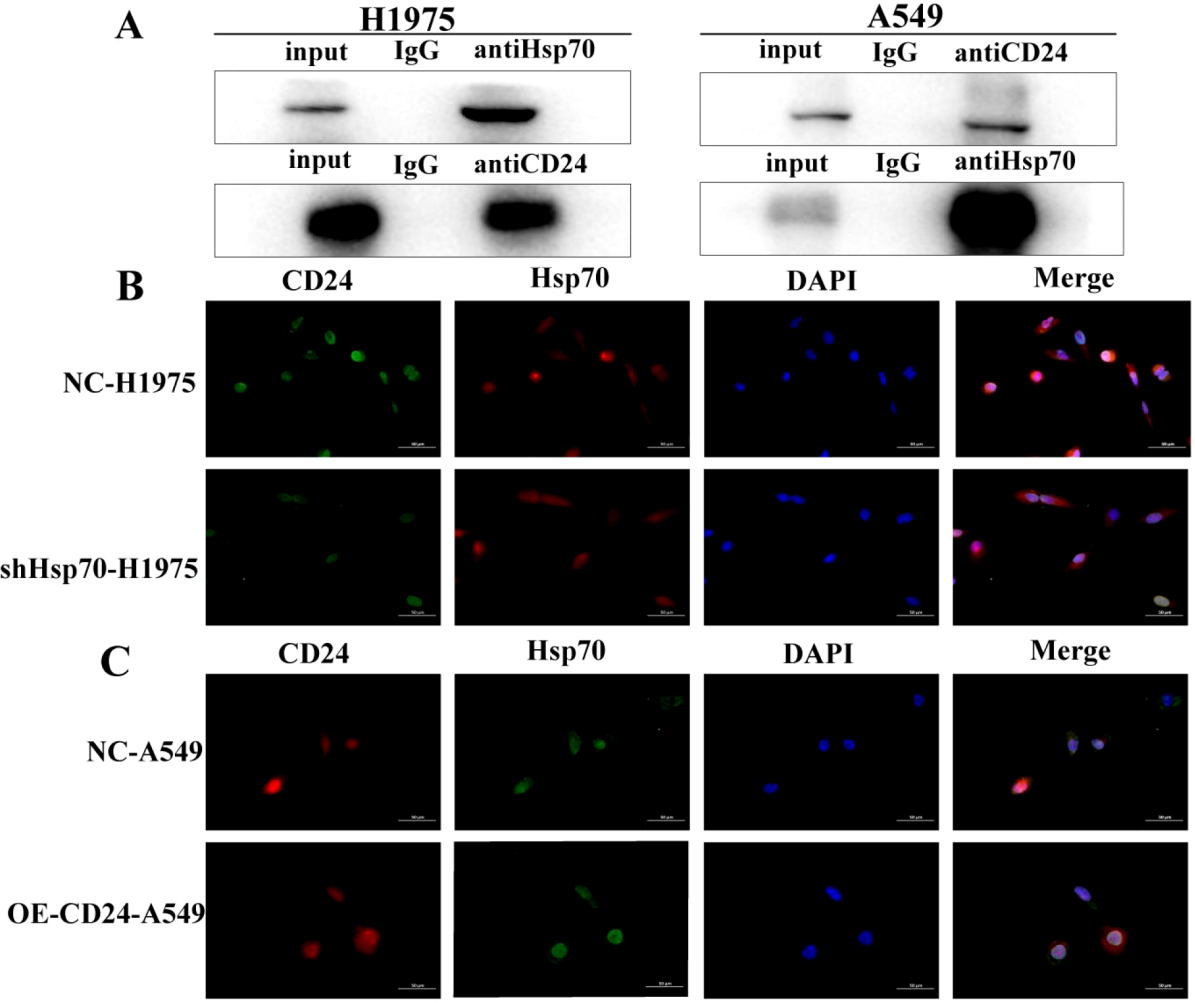
Co-IP confirms the interaction of Hsp70 with CD24. **(A)** In H1975 cells, bidirectional IP confirmed that Hsp70 co-precipitates with CD24; in A549 cells, Hsp70 co-precipitates with CD24 compared to control IgG. **(B, C)** Detection of protein interactions between CD24 and Hsp70 by immunofluorescence assay.

To characterize the molecular interaction between Hsp70 and CD24, we performed co-immunoprecipitation (co-IP) assays examining both endogenous and exogenous protein interactions. Bidirectional IP analysis in H1975 cells revealed robust co-precipitation of Hsp70 with endogenous CD24 (**Figure 4A**). Similarly, in CD24-overexpressing A549 stable transfectants, we observed significant Hsp70-CD24 complex formation compared to IgG controls. These findings demonstrate that both endogenous and recombinant CD24 physically associate with Hsp70, establishing their direct or indirect molecular interaction.

Immunofluorescence analysis revealed distinct subcellular colocalization of CD24 and Hsp70, with both proteins detected at the plasma membrane and within the cytoplasmic compartment (**Figures 4B, C**). Notably, Hsp70 knockdown in H1975 cells substantially reduced CD24 expression levels, whereas CD24 overexpression in A549 cells failed to modulate Hsp70 expression. These findings establish a unidirectional regulatory relationship, wherein Hsp70 functions as an upstream regulator of CD24 expression without reciprocal feedback regulation.

### 3.5 Hsp70 and CD24 affect the distant metastasis of lung cancer *in vivo*

**Figure 5:**
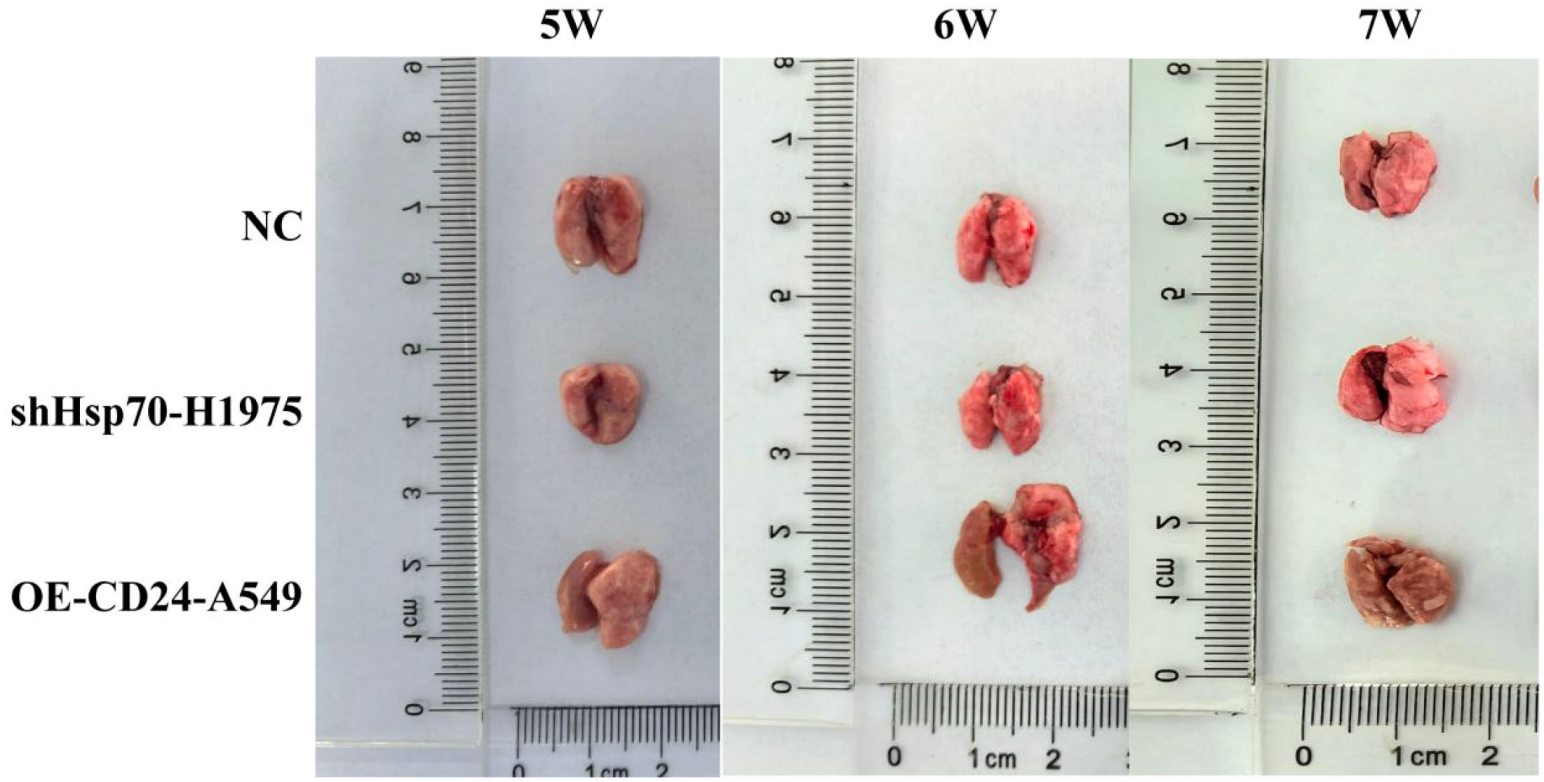
Lung metastases in mice at weeks 5–7 after tumor cell inoculation. NC: the mice injected with normal human lung epithelial cells (the non-malignant counterpart of our lung cancer cell lines).

To evaluate the functional roles of Hsp70 and CD24 in lung cancer metastasis, we established a xenograft model via tail vein injection in immunocompromised mice (n = 6 per group). All animals tolerated the procedure well, maintaining normal physiological parameters throughout the study period. Metastatic progression was monitored weekly. Week 5: No detectable metastases in either CD24 overexpression (OE-CD24-A549) or control groups; Week 6: OE-CD24-A549 group developed visible pulmonary nodules with diffuse left lung involvement; Week 7: Progressive metastasis observed, characterized by numerous translucent nodules and extensive parenchymal infiltration. Notably, Hsp70-knockdown (shHsp70-H1975) group showed complete absence of metastatic lesions in both pulmonary and extrapulmonary tissues, with no signs of pleural effusion (**Figure 5**). The results showed that CD24 overexpression promoted the distant metastasis of lung cancer in nude mice, while Hsp70 knockdown had the opposite effect.

### 3.6 The regulatory mechanism of Hsp70 and CD24 in lung cancer

**Figure 6:**
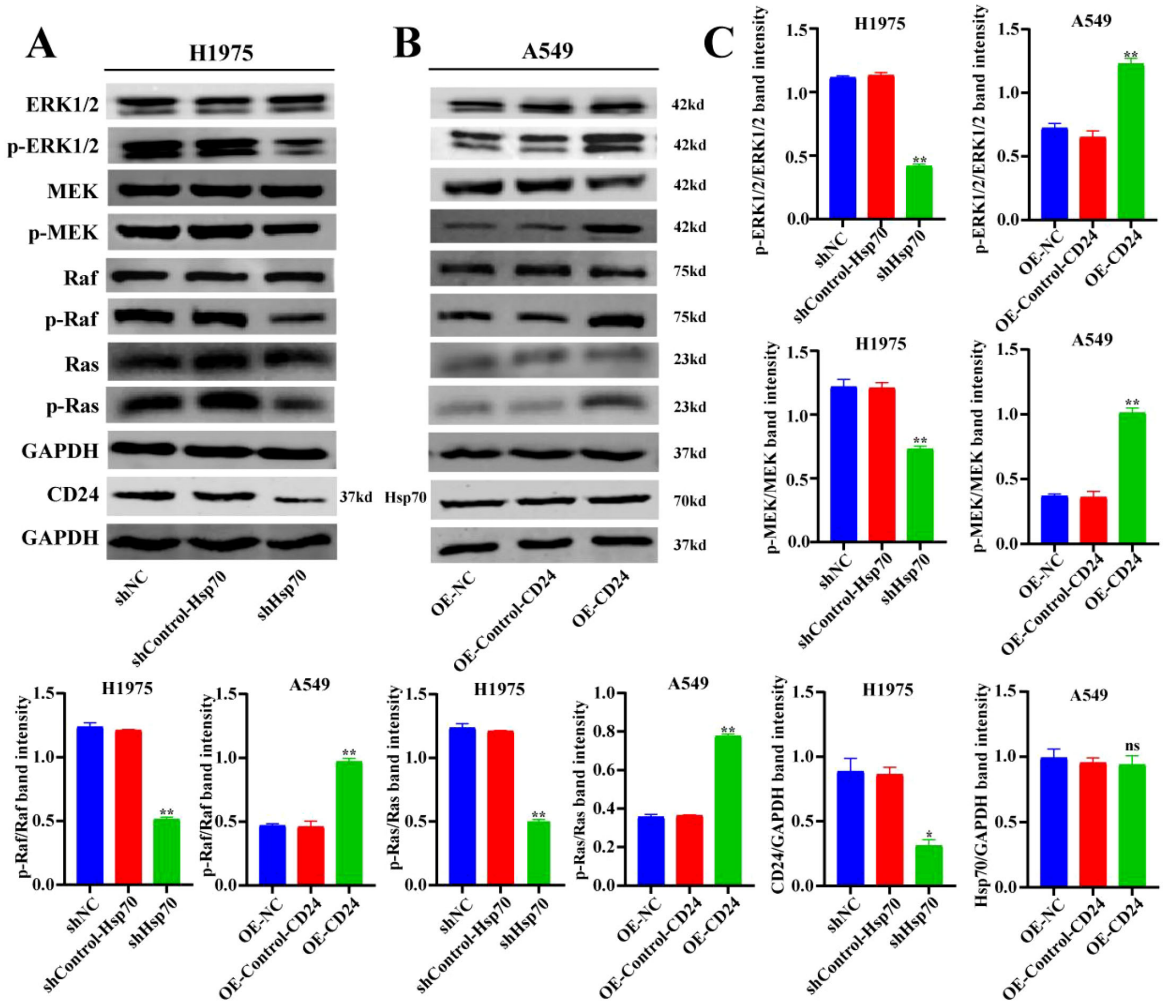
The regulatory mechanism of Hsp70 and CD24 in lung cancer. WB detection of MAPK/ERK signaling pathway-related protein levels. *P < 0.05, **P < 0.01.

We further studied the mechanism of the involvement of Hsp70 and CD24 in lung cancer. Through WB experiments, it was found that the expression levels of phosphorylated ERK1/2 were decreased in the shHsp70-H1975 group compared with the control group (p<0.01) (**Figure 6**), while they were increased in the OE-CD24-A549 group compared with the control group (p<0.01) (**Figure 6**). Western blot analysis revealed that the activation of ERK1/2, MEK, Raf, and Ras was significantly enhanced in the OE-CD24-A549 group compared to the control (p<0.01) (**Figure 6**), whereas it was markedly suppressed in the shHsp70-H1975 group (p<0.01) (**Figure 6**). Furthermore, the decreased expression of CD24 was verified after Hsp70 knockdown in H1975 cells (p<0.05) (**Figure 6C**), whereas there was no change in Hsp70 expression after CD24 overexpression in A549 cells (**Figure 6C**). This information confirms the positive correlation between the MAPK/ERK signaling pathway and the protein expression levels of Hsp70 and CD24.

## 4 Discussion

The discovery of the HSP70 family, known as the heat shock protein family, can be traced back to the laboratory of Ferruccio Ritossa in 1962. Over the next two decades, numerous studies from various countries further demonstrated that the primary function of these proteins was to protect cells from various nonlethal external stresses, particularly heat shock stimuli (22–24). On the basis of these research findings, this phenomenon of specific protein transcription increasing because of elevated temperature was described the heat shock response, and these proteins were termed heat shock proteins (25). A group of proteins with a molecular weight of approximately 70 kDa was classified as the HSP70 family. Subsequently, with the advancement of sequencing technologies, some genes with similar sequence structures were also included in the HSP70 family. Currently, there are 13 homologs in the HSP70 family in the human genome (26).

In this study, we found that Hsp70 is relatively overexpressed in lung cancer tissues and cells compared with normal lung tissues and cells. Such overexpression correlates with significantly enhanced proliferation, migration, and invasive capabilities of cancer tissues and cells compared with normal cells. However, the primary function of Hsp70 is as a molecular chaperone involved in regulating protein folding, protein translocation across membranes, protein complex degradation, and protein activity. Additionally, it maintains protein stability under stress conditions, including preventing protein aggregation and promoting disassembly, the refolding of misfolded proteins, and degradation (4). In this context, the dysregulation of Hsp70, especially its overexpression, should significantly enhance the function of heat shock proteins, making protein structures more stable and accelerating the refolding and degradation of misfolded proteins, thereby theoretically making it less likely that cancer cells and tissues would develop. In this study, we knocked down Hsp70 protein, which resulted in weakened proliferation, migration, and invasive abilities of lung cancer cells, further confirming the oncogenic role of overexpressed Hsp70. This effect extends beyond the recognized function of maintaining protein stability under stress, including preventing protein aggregation and promoting disassembly, the refolding of misfolded proteins, and degradation. Instead, overexpressed Hsp70 promotes the malignant biological behavior of lung cancer tissues and cells. However, this process is not yet fully understood. To investigate the underlying reasons for this phenomenon, we need to focus on another important function of Hsp70: its role as a client protein and molecular chaperone.

In the typical structure of Hsp70, the nucleotide-binding domain (NBD) is located at the N-terminus and consists of four subdomains (IA, IB, IIA, and IIB), arranged into two lobes separated by a deep cleft (4, 27). The primary function of the NBD is to bind and hydrolyze ATP to control the movement of the lobes (28). Subsequently, hydrophobic residues in the structure connect the NBD with the substrate-binding domain (SBD) at the C-terminus, which is crucial for the conformational changes in the NBD when ATP binds (29). The function of the SBD is closely related to the binding status of ATP/ADP to the NBD. When ATP binds to the NBD, interdomain linkers and SBDα/β collectively induce conformational changes in the NBD, rendering it unsuitable for ATP hydrolysis. When the NBD switches to the ADP-bound state, the SBD binds to the substrate with high affinity and a slow binding rate (30). Research has indicated that Hsp70 requires cooperation with other co-chaperones to complete its functional cycle (5, 27). The two most important co-chaperones are HSP40, also known as J-domain protein (JDP), and nucleotide exchange factor (NEF) (31). Generally, JDP transfers substrates to Hsp70 and stimulates the ATPase domain, while NEF induces substrate release and ATP rebinding. It is speculated that Hsp70 forms different protein complexes with its co-chaperones and various client proteins to exert different functions (32, 33).

Based on this principle, this study focuses on the CD24 protein. CD24, located on chromosome 6q21, is a highly variable glycosylphosphatidylinositol-anchored membrane protein. In 1978, Springer et al. discovered a heat-stable, organic solvent-soluble glycoprotein with a lipophilic structure on the surface of mouse leukocytes and named it HAS (34). In 1991, Kay et al. confirmed that CD24 is the human homolog of HAS. CD24 consists of 27 amino acids and is a highly glycosylated adhesion molecule anchored to the cell membrane by glycosylphosphatidylinositol (35); it is further tethered to lipid rafts within the cell membrane, where it functions in transmembrane signal transduction. Recent studies have shown that CD24 is overexpressed in various human tumors, including liver cancer, thyroid cancer, and esophageal cancer, where it plays a crucial role in tumor initiation, progression, invasion, and metastasis.

Through previous studies, our research group has confirmed that CD24 is an independent prognostic risk factor for lung cancer patients (3). In this study, we further confirmed the interaction between Hsp70 and CD24 both *in vivo* and *in vitro*. CD24 is one of the client proteins of Hsp70, forming protein complexes with it to promote the invasion and metastasis of lung cancer. Previous studies have shown that Hsp70 promotes the interaction between MEK1/2 and PP1α, facilitating the PP1α-mediated dephosphorylation of MEK1/2 in an ATP-sensitive manner (36). However, whether it directly or indirectly affects ERK remains unclear. Therefore, this study validated this process and found that knocking down Hsp70 led to a decrease in the levels of total ERK1/2 and phosphorylated ERK1/2, while overexpression of CD24 resulted in an increase in the levels of total ERK1/2 and phosphorylated ERK1/2. The MAPK/ERK signaling pathway directly participates in processes such as cell proliferation, differentiation, and apoptosis. This research aligns with the theoretical results proposed by Wang et al. in 2010 (37), who suggested that CD24 may directly or indirectly activate the MAPK signaling pathway to promote tumor invasion and metastasis.

## 5 Conclusions

This study identifies HSP70 as a critical upstream regulator of CD24 in lung cancer, demonstrating a unidirectional regulatory relationship wherein HSP70 controls CD24 expression without reciprocal feedback. The binding of Hsp70 to CD24 promotes the invasion and metastasis of lung cancer through the MAPK/ERK signaling pathway. Clinically, the HSP70-CD24 interaction serves both as a prognostic biomarker and a promising therapeutic target, offering novel avenues for intercepting lung cancer metastasis.

## 6 Limitations of the study

This study has some important limitations. For instance, the specific binding mode and mechanism of action between Hsp70 and CD24 have not been confirmed, the correlation between other possible client proteins of Hsp70 and CD24 was not studied, and the investigation of signaling pathways and mechanisms was not comprehensive enough. These areas will be the focus of future research by our research group.

## Data Availability

The raw data supporting the conclusions of this article will be made available by the authors, without undue reservation.
