# Supplementary material for: Hsp70 regulates CD24 expression and promotes metastasis and invasion of lung cancer via the MAPK/ERK signaling pathway
Source: Front Oncol. 2025 Oct 21;15:1665342. doi: 10.3389/fonc.2025.1665342 (PMC12583058; doi:10.3389/fonc.2025.1665342)
Supplement: Supplementary file 1 [file Table1.docx]

Supplementary Table 1: Primer information

| CD24 Forward primer | 5'-TTCTCCAAGCACCCAGCA-3' |
| --- | --- |
| CD24 Reverse primer | 5'-TGGAATAATCTGCGTGGGTA-3' |
| Hsp70 Forward primer | 5'-GGGCCTTTCCAAGATTGCTGT3' |
| Hsp70 Reverse primer | 5'-ATCTCTGCATGTAGAAACCGGAAA3' |
| GAPDH Forward primer | 5’-GGAGCGAGATCCCTCCAAAAT-3 ’ |
| GAPDH Reverse primer | 5'-GGCTGTTGTCATACTTCTCATGG-3' |
