# Supplementary material for: Hsp70 regulates CD24 expression and promotes metastasis and invasion of lung cancer via the MAPK/ERK signaling pathway
Source: Front Oncol. 2025 Oct 21;15:1665342. doi: 10.3389/fonc.2025.1665342 (PMC12583058; doi:10.3389/fonc.2025.1665342)
Supplement: Supplementary file 2 [file Table2.docx]

Supplementary Table 2: Antibody information

| **Antibody** | **Dilution** | **Company** | **catalog** |
| --- | --- | --- | --- |
| GAPDH | 1:20000 | Proteintech | 60004-1-Ig |
| Beta Tubulin | 1:6000 | Proteintech | 10094-1-AP |
| HSP70 | 1:2000 | Abmart | T55496S |
| CD24 | 1:5000 | Proteintech | 67627-1-Ig |
